# Plumbagin Modulates Leukemia Cell Redox Status

**DOI:** 10.3390/molecules190710011

**Published:** 2014-07-10

**Authors:** François Gaascht, Marie-Hélène Teiten, Claudia Cerella, Mario Dicato, Denyse Bagrel, Marc Diederich

**Affiliations:** 1 Laboratoire de Biologie Moléculaire et Cellulaire du Cancer (LBMCC), Hôpital Kirchberg, 9, Rue Edward Steichen, L-2540 Luxembourg, Grand-Duchy of Luxembourg; E-Mails: francois.gaascht@lbmcc.lu (F.G.); marie_helene.teiten@lbmcc.lu (M.-H.T.); claudia.cerella@lbmcc.lu (C.C.); mdicato@gmail.com (M.D.); 2 Laboratoire Structure et Réactivité des Systèmes Moléculaires Complexes, UMR CNRS 7565, Université de Lorraine, Campus Bridoux, Rue du Général Delestraint, F-57070 Metz, France; E-Mail: denyse.bagrel@univ-lorraine.fr; 3 Department of Pharmacy, College of Pharmacy, Seoul National University, Seoul 151-742, Korea

**Keywords:** plumbagin, natural products, leukemia, cancer, reactive oxygen species, glutathione, apoptosis, thiol groups

## Abstract

Plumbagin is a plant naphtoquinone exerting anti-cancer properties including apoptotic cell death induction and generation of reactive oxygen species (ROS). The aim of this study was to elucidate parameters explaining the differential leukemia cell sensitivity towards this compound. Among several leukemia cell lines, U937 monocytic leukemia cells appeared more sensitive to plumbagin treatment in terms of cytotoxicity and level of apoptotic cell death compared to more resistant Raji Burkitt lymphoma cells. Moreover, U937 cells exhibited a ten-fold higher ROS production compared to Raji. Neither differential incorporation, nor efflux of plumbagin was detected. Pre-treatment with thiol-containing antioxidants prevented ROS production and subsequent induction of cell death by apoptosis whereas non-thiol-containing antioxidants remained ineffective in both cellular models. We conclude that the anticancer potential of plumbagin is driven by pro-oxidant activities related to the cellular thiolstat.

## 1. Introduction

Development of anti-cancer drug resistance and differential susceptibility of patients remain the main factors reducing the effectiveness of current chemotherapeutic treatments. Combinational therapies and the identification of novel potent and specific anti-cancer agents could contribute to improve existing therapies. However, it has become clear that a personalized approach could be the key to more effective treatments. In this view, correlative studies linking cancer patients’ genetic or epigenetic [1,2,3] background to treatment response will be crucial for both therapeutic outcome and elucidation of unknown molecular mechanisms to eventually achieve “targeted therapies”.

Natural compounds, whether extracted from terrestrial or marine organisms, offer a large variability of molecular scaffolds with anti-cancer potential. Frequently, they derive from medicinal or dietary traditions that already provided health-promoting effects for centuries [4,5,6,7]. Amongst the best-known plant compounds, polyphenols like curcumin [8,9,10,11] or polysulfides [12,13,14,15], cardiac glycosides [16,17,18] were recently investigated and corresponding anticancer mechanisms were identified. Moreover interesting anti-cancer compounds from entophytic fungi were recently investigated [19,20].

Plumbagin (5-hydroxy-2-methyl-1,4-naphthoquinone) is a secondary metabolite produced by various plants (*Plumbago* *zeylanica*, *Dionaea* *muscipula*, *Nepenthes* *gracilis*, *Drosera* *binata* *or* *Juglans* *regia*) [19,21,22] and acts as a potent anti-cancer agent in various cellular cancer models, including breast, cervical, gastric, lung, melanoma, prostate cells [23,24,25,26,27,28]. A number of studies have shown that plumbagin acts on cancer cells as cell cycle inhibitor [24,28,29], cytotoxic agent [27,30,31,32,33,34], angiogenesis inhibitor [35,36], and as a modulator of various cancer-specific pathways (*i.e.*, mediated by NF-κB [37,38] or mitogen activated protein kinases (MAPK) [28,29,39]. Mechanistic studies suggest that the anti-cancer effects of plumbagin depend mostly on its ubiquitous pro-oxidant activities. Accordingly, plumbagin elicits intracellular ROS in a number of cancer cell models; moreover, strategies preventing or scavenging ROS formation inhibit the biological effects ascribed [40,41,42,43,44]. Glutathione (GSH), the major cellular anti-oxidant, was identified as a direct target of plumbagin by its ability to bind GSH [22,45]. Indirectly, it has been suggested that plumbagin acts as an electrophile against GSH [46]. Finally, plumbagin was shown to inhibit glutathione-*S*-transferase (GST) [47,48]. Altogether, these findings suggest that plumbagin regulates the cellular redox state by modulation of GSH even though additional redox-dependent mechanisms could be directly or indirectly involved.

The favorable differential effect of plumbagin on cancer *vs*. healthy cell models and the confirmation of its effectiveness in *in* *vivo* experimental models suggest plumbagin as a promising candidate for more advanced investigations. However, further elucidation of its mechanism of action is still required especially to identify the most susceptible cancer cell models.

In this study, we analyzed the effect of plumbagin on the viability of a panel of human hematopoietic cancer cell models, including chronic and acute forms of hematological malignancies (U937, Raji, K562, Jurkat, HL-60) compared to peripheral blood mononuclear cells (PBMCs) from healthy donors. We selected the U937 (most sensitive) and Raji (less sensitive) cells for a comparative mechanistic study. As PBMCs were not affected by the treatment, we also confirmed the excellent differential anti-cancer potential of plumbagin. Altogether, we observed that the pro-oxidant regulation is independent of a differential intake/uptake of the compound by the two cancer cell models. We rather demonstrate the differential ability of the compound to elicit ROS in U937 and Raji cells as well as to impact the intracellular GSH pool. We finally suggest a differential expression of redox-related factors as potential regulators of the observed differential susceptibility.

## 2. Results and Discussion

### 2.1. Plumbagin Reduces Leukemia Cell Viability

Evaluation of the effect of plumbagin on the viability of different leukemia cell lines by trypan blue exclusion assay revealed that this compound presents a cytotoxic effect towards all cell lines tested (Table 1). U937 cells appear as the most sensitive cell line with an IC_50_ ranging from 0.82 ± 0.04 μM to 0.66 ± 0.02 μM observed between 24 and 72 h of treatment. Raji cells were less sensitive with an IC_50_ value of 5.06 ± 0.22 μM and 2.66 ± 0.03 μM respectively after 24 and 72 h treatment. Even at the highest concentration tested (10 μM), PBMCs were not affected by plumbagin treatment. For further mechanistic studies of the effects of plumbagin, we selected U937 and Raji cells to perform a comparative analysis using IC_50_ concentrations at 24 h, respectively, 1 µM for U937 and 5 µM for Raji cells.

**Table 1:** Cytotoxic effect of plumbagin on different human leukemia cell lines compared to PBMCs from healthy donors. IC_50_ values were determined by three independent trypan-blue assays after 24, 48 and 72 h of treatment. The data are the mean of at least three independent experiments ± SD. N.C. stands for “not cytotoxic” (viability > 80%) for a concentration up to 10 μM.

Cell Lines	IC_50_ (µM)		
	24 h	48 h	72 h
**HL-60**	1.38 ± 0.37	0.92 ± 0.16	0.90 ± 0.13
**Jurkat**	2.20 ± 1.07	0.98 ± 0.15	0.86 ± 0.16
**K562**	1.07 ± 0.33	0.90 ± 0.32	0.89 ± 0.30
**Raji**	5.06 ± 0.22	3.49 ± 0.12	2.66 ± 0.03
**U937**	0.82 ± 0.04	0.68 ± 0.01	0.66 ± 0.02
**PBMC**	N.C.	N.C.	

### 2.2. Plumbagin Induces Apoptotic Cell Death

**Figure 1:**
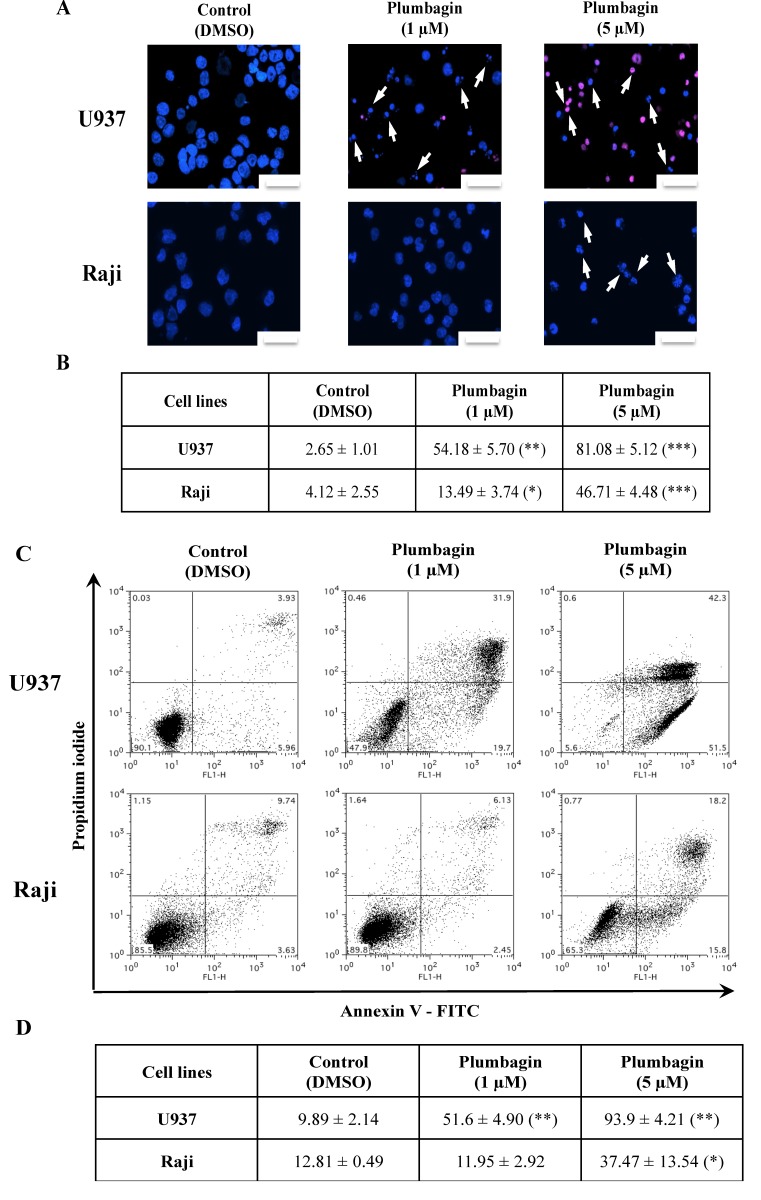
Cell death was assessed in U937 and Raji cells treated with plumbagin for 24 h at concentrations corresponding to their respective IC_50_ values. (**A**,**B**) Double staining with Hoechst and propidium iodide (PI) (the images shown are representative for at least three independent experiments); scale at the lower right corner = 100 μm. Arrows point to cells showing apoptotic features after plumbagin treatment; (**C**,**D**) Annexin V/PI staining and flow cytometry analysis (one of three experiments shown). Cell population corresponding to early and late (in secondary necrosis) apoptotic cells are respectively in the lower and upper right quadrants. All results presented are the mean ± SD of at least three independent experiments. The values in both tables correspond to percentage of apoptotic cells of at least three independent experiments. * *p* *<* 0.05, ** *p* *<* 0.01 and *** *p* *<* 0.001 compared to non-treated cells, respectively.

**Figure 2:**
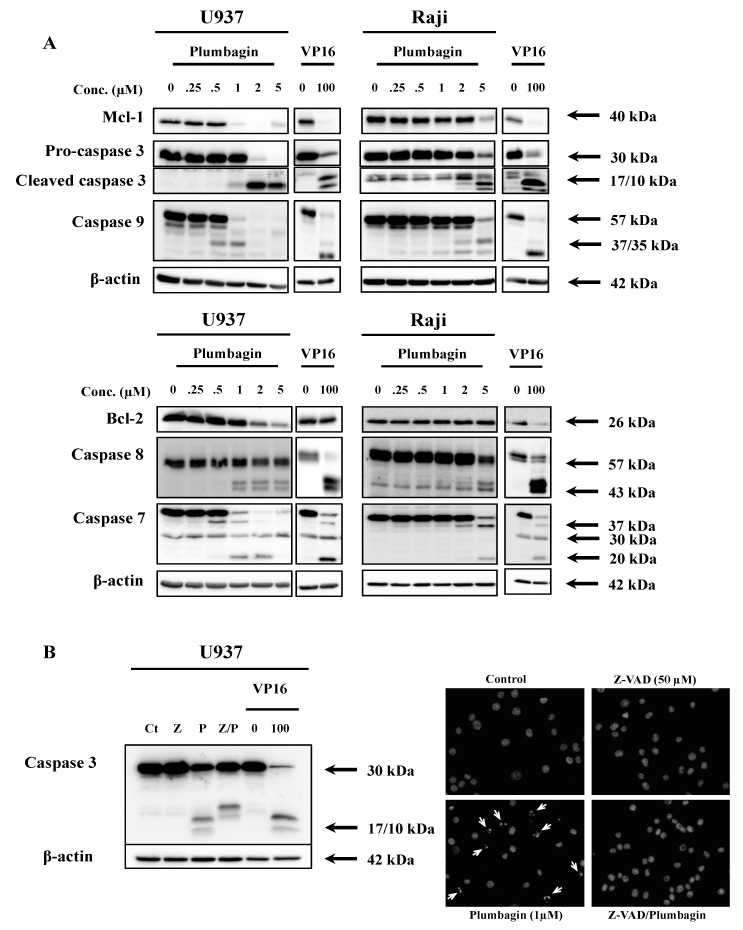
Effect of plumbagin on pro- and anti-apoptotic cell markers. (**A**) Caspase activation and expression of anti-apoptotic Mcl-1 and Bcl-2 markers were analyzed by Western-Blot after 16 h of treatment by plumbagin; (**B**) Pre-treatment with Z-VAD-FMK (50 μM), a pan-caspase activity inhibitor. Western Blot analysis (left panel); fluorescence microscope observations upon double staining with Hoechst. Arrows point to cells showing apoptotic features after plumbagin treatment (right panel). C: control, Z: Z-VAD-FMK, P: plumbagin (1 μM), Z/P: cells pre-treated with Z-VAD-FMK and then treated with plumbagin (1 μM). U937 cells treated with VP16 were used as apoptosis positive control. Results are representative of at least three independent experiments.

Considering the elevated levels of cytotoxicity, we analyzed the type of cell death triggered by plumbagin in U937 and Raji cells by fluorescence microscopy after staining with Hoechst and propidium iodide (PI). 24 h of treatment at a concentration of 1 μM (U937) and 5 μM (Raji) induced the appearance of nuclear morphological alterations compatible with apoptosis in both cell lines (Figure 1A,B). This finding was further confirmed by the analysis of the exposure of phosphatidylserine by Annexin V/PI assay (Figure 1C,D). Results pointed out that both cell lines died by an apoptotic process in a dose-dependent manner. These results were confirmed by Western-blot analysis that showed caspase cleavage and decrease Mcl-1 and Bcl-2 anti-apoptotic protein expression levels, starting from the respective IC_50_ concentration of plumbagin in U937 and Raji cells (Figure 2A). Pre-treatment with the pan-caspase activity inhibitor (Z-VAD-FMK) prevented the 17–10 kDa caspase-3 fragment formation. This result confirms that plumbagin induces cell death by a caspase-dependent apoptotic process (Figure 2B). These results have been confirmed by fluorescence microscopy analysis after Hoechst staining (data not shown).

### 2.3. Plumbagin Induces Different Levels of Intracellular ROS in U937 vs. Raji Cells

**Figure 3:**
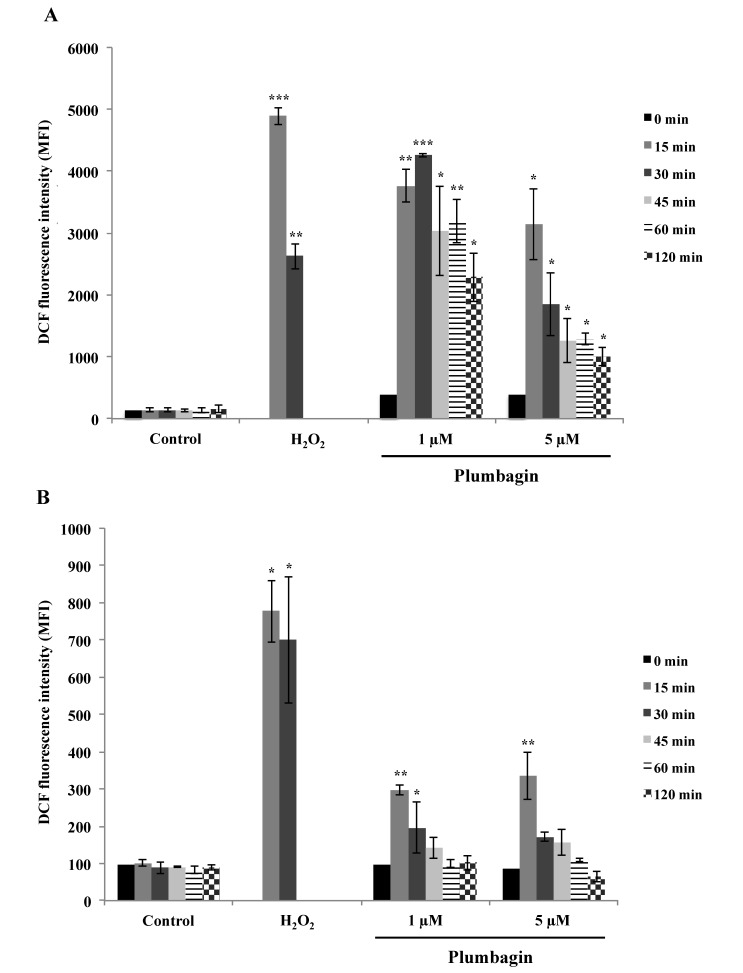
Time-course analysis of ROS measured in U937 (**A**) and Raji (**B**) cells upon treatment with plumbagin was assessed with 2',7'-dichlorodihydrofluorescein diacetate (H_2_DCFDA). Results are the mean ± SD of at least three independent experiments. * *p* *<* 0.05, ** *p* *<* 0.01 and *** *p* *<* 0.001 compared to non-treated cells, respectively.

Published data demonstrated the capacity of plumbagin to elicit ROS in cancer cells [26,27,49]. Analysis of intracellular ROS production in U937 and Raji cells exposed to plumbagin was performed by flow cytometry analysis after staining with 2',7'-dichlorodihydrofluorescein diacetate (H_2_DCFDA). As shown in Figure 3A,B, plumbagin induces ROS production in both cell lines tested. U937 cells show a very robust increase in intracellular ROS already after 15 min treatment at 1 μM plumbagin. Raji cells, in contrast, show a much milder intracellular ROS increase compared to the positive control hydrogen peroxide (H_2_O_2_, 50 μM). The level of ROS production induced by plumbagin is ten-fold higher in U937 compared to Raji cells. The incubation of Raji cells with a lower, sub-apoptogenic, concentration of plumbagin (1 μM) showed a comparable level of ROS production. The flow cytometry analysis did not reveal any cell subpopulations differently responsive to ROS production, therefore indicating a homogenous ability of cells to increase ROS production upon treatment. Moreover, no significant changes in ROS production were observed for longer incubation times with both cell lines. These results suggest that the differential plumbagin-induced ROS production is an early event.

### 2.4. Differential ROS Generation Is not a Consequence of a Different Uptake/Efflux of Plumbagin

**Figure 4:**
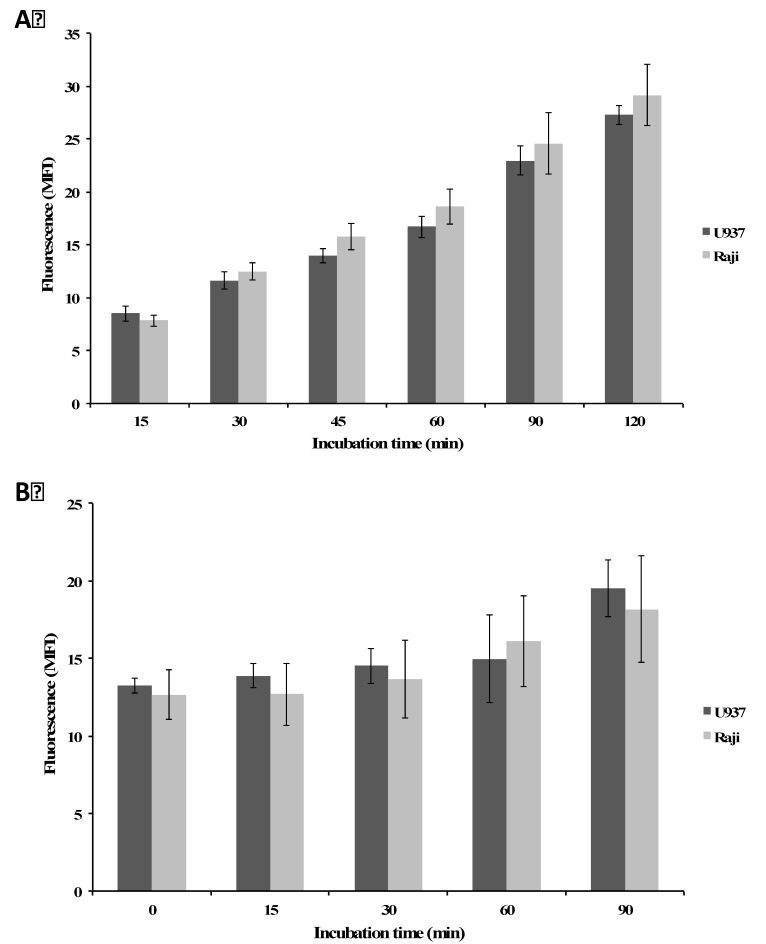
(**A**) Plumbagin uptake assay was performed after treatment of cells at 5 μM. Samples were collected and analyzed by flow cytometry without additional staining; (**B**) Plumbagin efflux assay was performed after 30 min of incubation with the same concentration of plumbagin used for the uptake assay, followed by recovery in plumbagin-free medium (see “Experimental” section) [50]. U937 and Raji cells were collected at indicated times and their auto-fluorescence was analyzed by flow cytometry. Untreated cells did not show any modulation of fluorescence over the time as expected (data not shown). Results are the mean ± SD of at least three independent experiments.

The observed ROS generation could be the consequence of a differential internalization of plumbagin. As plumbagin is a fluorescent pigment [45], we then investigated differential internalization. Analysis of the intracellular fluorescence of plumbagin by flow cytometry (see “Experimental” section) revealed that this compound accumulates similarly in U937 and Raji cell lines as a function of incubation time (Figure 4A). Besides, in the drug-efflux test, no decrease of the plumbagin-generated intracellular fluorescence was observed in both cell lines up to 90 min of incubation in plumbagin-free culture medium (recovery). Fluorescence maintained very high levels up to 4 h (not shown) without any difference depending on the cell models. Untreated cells did not show any modulation of fluorescence over the time as expected (data not shown). This aspect may reveal specific aspects related to the intracellular metabolism of the compound and may deserve future investigations, beyond the scope of this study. Altogether, we can exclude a differential internalization or efflux of the compound as the responsible factor for lower ROS levels in Raji.

### 2.5. Thiol-Containing Antioxidants Prevent Plumbagin-Induced Apoptosis

**Figure 5:**
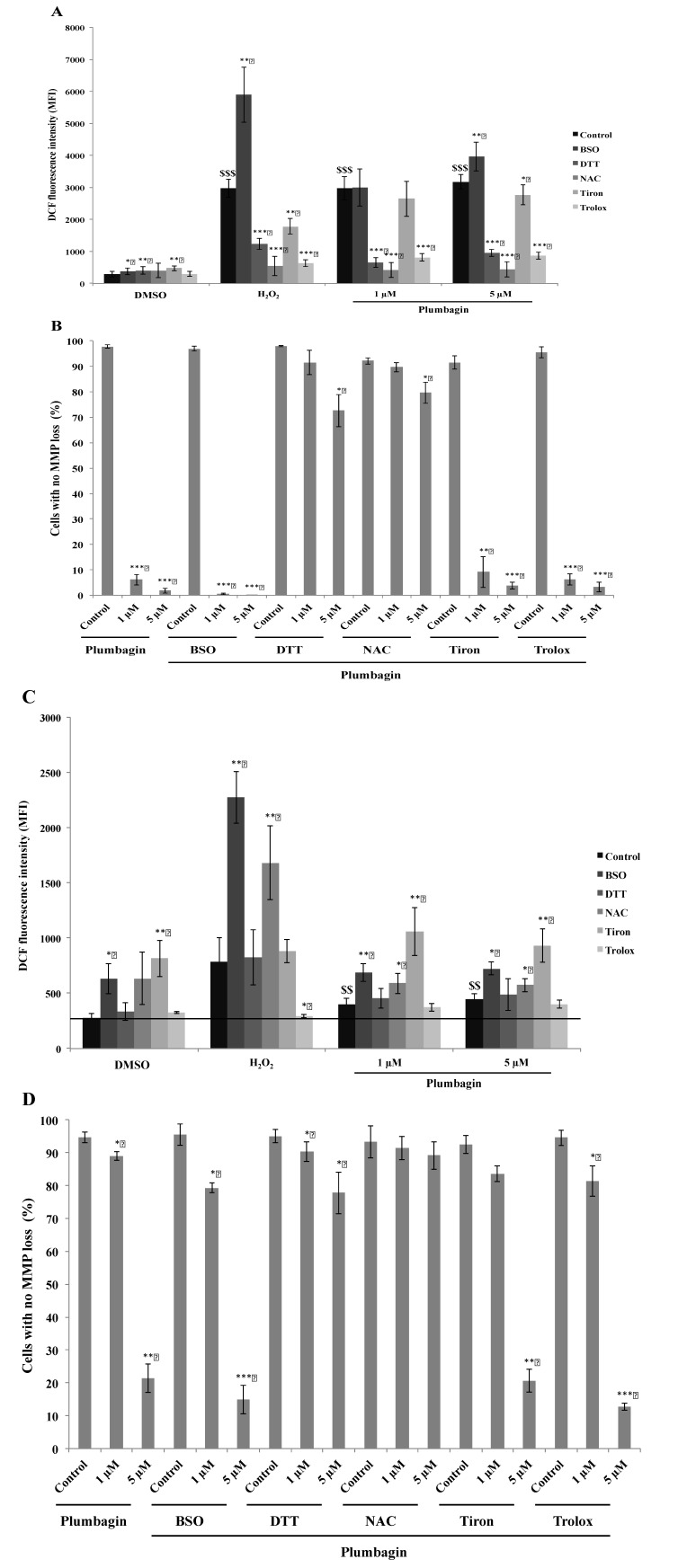
(**A**) ROS measurement in U937 after 15 min of treatment with plumbagin using H_2_DCFDA. Cells were pre-treated with BSO and different antioxidants (DTT, NAC, Tiron and Trolox) prior to ROS analysis by flow cytometry; (**B**) Mitochondrial membrane potential was determined in U937 cells 24 h after plumbagin treatment; (**C**) ROS measurement in Raji cells was performed under the same conditions as used for U937 cells (see point (**A**)); (**D**) Mitochondrial membrane potential was determined in Raji cells 24 h after plumbagin treatment. Results are the mean ± SD of at least three independent experiments. For the statistical analysis, results were considered as statistically significant for *p* values * *p* *<* 0.05, ** *p* *<* 0.01 and *** *p* *<* 0.001 when compared to their respective control (black bars for the ROS measurement analysis), and were considered as statistically significant for *p* values ^§§^ *p* <0.01 and ^§§§^ *p* <0.001 when compared to cells treated only with the vehicle DMSO.

As ROS are known to play a key role in apoptosis induction [51], we investigated the effect of antioxidants on ROS generation and apoptotic cell death induction. Using BSO, a GSH depletor, and H_2_O_2_ as positive controls for ROS generation, our analysis showed that the pre-treatment with DTT [52], NAC [12,15] and Trolox [53] buffers plumbagin-dependent ROS production whereas the metal chelator Tiron [53,54], a hydroxyl radical and superoxide scavenger, remained ineffective as antioxidant agent in both models (Figure 5A,C). Then, we estimated the percentage of apoptosis by analyzing the loss of mitochondrial membrane potential (see “Experimental” section), a marker of the mitochondrial apoptotic pathway [12,50]. Only pre-treatment with DTT or NAC, two thiol-containing antioxidants, prevented cell death, while non-thiol antioxidants, Tiron and Trolox, did not affect apoptosis, although Trolox was able to buffer ROS formation (Figure 5B,D). Cancer cells typically develop alterations of their oxidative status, by showing altered expression patterns of enzymes whose function might depend on thiol modulation [40,42,43,55,56,57]. Our findings indicate an ability of plumbagin to eventually lead to the modulation of important intracellular functions especially those dominated by thiol modulation, which include also enzymes controlling and/or modulating the cellular redox status in cancer cells.

### 2.6. Plumbagin Decreases the Intracellular GSH Level

**Figure 6:**
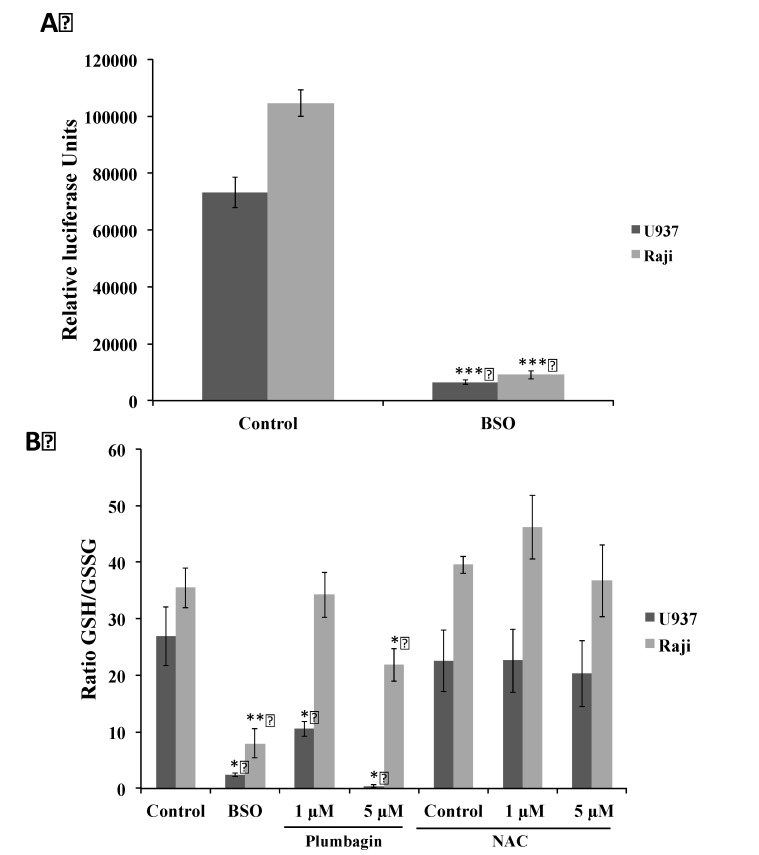
(**A**) Total glutathione levels (GSH + GSSG) were determined in U937 and Raji cells using a GSH/GSSG-Glo Assay (Promega). Cells pre-treated during 24 h with BSO (1 mM) were used as control; (**B**) GSH/GSSG ratios of U937 and Raji cells were measured after 1 h of treatment with plumbagin. Cells pre-treated during 24 h with BSO (1 mM) or NAC (10 mM) were used as control. Results are the mean ± SD of at least three independent experiments. * *p* *<* 0.05, ** *p* *<* 0.01 and *** *p* *<* 0.001 compared to non-treated cells, respectively.

Next, we investigated the impact of plumbagin on GSH as it has been shown that GSH depletion is a common feature in apoptotic cell death [58]. To elucidate the mechanisms explaining the differential sensitivity, we measured the level of total GSH, which is 30% higher in Raji compared to sensitive U937 cells (Figure 6A). These differential GSH levels could explain the plumbagin-induced ROS production observed in the two selected models (Figure 3 and Figure 5). Then, we analyzed the GSH/GSSG ratio after stimulation with plumbagin using BSO- and NAC-treated cells as controls. Plumbagin decreases the GSH/GSSG ratio in both cell lines in a dose-dependent manner. NAC treatment per se did not significantly alter the GSH/GSSG ratio observed in control cells.

In Raji cells, a 40% decrease is observed at 5 μM compared to a decrease of 60% in U937 cells (Figure 6B). The analysis of reduced GSH by *O*-phthalaldehyde (OPA) assay revealed that a dose of 5 μM of plumbagin is requested to decrease the GSH content by 25% in Raji cells whereas 1 μM is sufficient to reach the same level in U937 cells. 24 h pre-treatment with NAC completely abrogates the previously described depletion of GSH by plumbagin (Table 2).

**Table 2:** Intracellular GSH level was determined using OPA probe after 1 h of treatment with plumbagin. Cells pre-treated during 24 h with NAC (10 mM) were used as control. Results are the mean ± SD of at least three independent experiments (values are indicated as a ratio of fluorescence values between treated and control cells). * *p* *<* 0.05, and ** *p* *<* 0.01 compared to non-treated cells, respectively.

Treatment	Cell Lines	Control	H_2_O_2 _(50 µM)	Plumbagin (1 µM)	Plumbagin (5 µM)
**Control** (DMSO)	U937	100.00	88.38 ± 17.87	67.34 ± 9.21 (*)	66.20 ± 13.26 (*)
	Raji	100.00	94.10 ± 13.88	88.49 ± 16.93	80.38 ± 5.59 (*)
**NAC** (10 mM)	U937	100.00	89.99 ± 6.42	92.59 ± 17.54	85.71 ± 3.86 (**)
	Raji	100.00	111.36 ± 11.02	110.76 ± 8.74	90.85 ± 14.80

## 3. Experimental

### 3.1. Reagents

Plumbagin (Sigma-Aldrich, Bornem, Belgium) was dissolved in dimethyl sulfoxide (DMSO) (Sigma-Aldrich) at a concentration of 100 mM. Subsequent dilutions were made in cell culture medium. Buthionine sulfoximine (BSO), N-acetyl-L-cysteine (NAC), H_2_O_2_, propidium iodide (PI) and Trolox were purchased from Sigma. Z-VAD-FMK (Calbiochem, San Diego, CA, USA). Tiron was purchased from Alfa Aesar (Karlsruhe, Germany) and dithiothreitol (DTT) from Roche (Prophac, Luxembourg). Dichlorofluorescein diacetate (H_2_DCFDA), and *O*-phthalaldehyde (OPA) probes were purchased by Life technologies Invitrogen (ThermoFisher, Alost, Belgium).

### 3.2. Cell Culture

HL-60 (human promyelocytic leukemia), Jurkat (T-cell leukemia), K562 (human chronic myelogenous leukemia), Raji (Burkitt lymphoma) and U937 (histiocytic lymphoma) cells were cultured in RPMI 1640 medium (Lonza, Verviers, Belgium) supplemented with 10% (*v/v*) fetal calf serum (FCS) (Lonza) and 1% (*v/v*) antibiotic-antimycotic (BioWhittaker, Verviers, Belgium) at 37 °C and 5% of CO_2_, humidified atmosphere. Exponentially growing cells were used for plumbagin treatment. Cells treated with DMSO (1 ‰) were used as control. For specific experiments, Raji and U937 cells were pre-treated with NAC (10 mM) or BSO (1 mM) for 24 h, DTT (100 μM) for 2 h, or with Tiron (10 mM) or Trolox (1 mM) for 1h before plumbagin treatment. Control cells were not pre-treated. Z-VAD-FMK (50 μM, 1 h)

Healthy blood samples were kindly donated as buffy coats by the Red Cross (Luxembourg, Grand Duchy of Luxembourg). By applying diluted (1/3) blood onto a Ficoll layer (GE Healthcare, Diegem, Belgium) followed by centrifugation (500 g, 30 min), mononuclear cells were isolated and collected. The isolated peripheral blood mononuclear cells (PBMCs) were cultured at 37 °C and 5% CO_2_ for 24 h before use.

### 3.3. Cell Viability Assay

Cancer cells (2 × 10^5^ cells/mL) and PBMCs (2 × 10^6^ cells/mL) were incubated with different concentrations of plumbagin during 24, 48 or 72 h. Cell viability was assessed by trypan blue exclusion test.

### 3.4. Fluorescence Microscopy

After plumbagin treatment, cells were stained with Hoechst 33342 (Calbiochem) and propidium iodide (Sigma–Aldrich) during 20 min at 37 °C. Labeled cells were analyzed with an inverted Cell M Olympus Microscope (Olympus, Aartselaar, Belgium) and Cell M software.

### 3.5. Apoptosis Assays

Apoptosis was assessed and estimated by three different assays: (1) analysis of nuclear fragmentation (Hoechst staining and fluorescence microscope observation, performed as previously described [59]; (2) evaluation of phosphatidylserine exposure and (3) evaluation of mitochondrial membrane potential. Evaluation of phosphatidylserine exposure was performed with the Annexin V: FITC Apoptosis Detection Kit I (BD Pharmingen, Erembodegem, Belgium) according to the manufacturer’s instructions. Briefly, after 24 h of treatment with different concentrations of plumbagin, 1 × 10^6^ cells were washed with cold phosphate buffered saline (PBS), resuspended in binding buffer and stained with Annexin V-FITC and propidium iodide for 15 min. To evaluate the reduction of mitochondrial membrane potential, plumbagin-treated cells were stained with 50 nM MitoTracker Red CMXRos (Invitrogen) during 20 min at 37 °C according to the manufacturer’s protocol. For both approaches, stained samples were analyzed by flow cytometry (FACSCalibur, BD Biosciences, San Jose, CA, USA). Data were recorded using CellQuest and further analyzed by FlowJo software version 8.8.7 (Tree Star Inc, Ashland, OR, USA) available online: http://www.flowjo.com.

### 3.6. Western-Blot

Total proteins extracts were subjected to sodium dodecyl sulfate polyacrylamide gel electrophoresis (SDS–PAGE, 12%) and transferred onto nitrocellulose membranes (Hybond™-P membrane, GE Healthcare). Membranes were pre-hybridized with 5% non-fat milk in PBS 1X containing 0.1% (*v/v*) Tween 20 (PBS-T) overnight at 4 °C or 1 h at room temperature. Membranes hybridizations with primary antibodies directed against caspase-3, caspase-7, caspase-8, caspase-9 (Cell Signaling, Bioké, Leiden, The Netherlands), Bcl-2 (Calbiochem) and β-actin (Sigma) used as loading control, were carried out in PBS-T containing 5% milk or 5% bovine serum albumin (BSA) for 1 h at room temperature or overnight at 4 °C, according to the providers’ protocols. Etoposide-treated U937 cells (VP16, 100 μM, 4 h) were used as apoptosis positive control and equal loading of samples was controlled using β-actin. After incubation with primary antibodies, membranes were washed and probed with the corresponding secondary (horseradish peroxidase conjugated) antibodies following manufacturers’ instructions for 1 h at room temperature. Proteins of interest were visualized with ECL Plus Western Blotting Detection Reagents (GE Healthcare) using the ImageQuant LAS 4000 Mini (GE Healthcare).

### 3.7. Evaluation of ROS Production

Raji and U937 cells were treated with plumbagin during 15, 30, 45, 60 or 120 min. 20 min before the end of the treatment, cells were stained at 37 °C with 10 μM of 2',7'-dichlorodihydrofluorescein diacetate (H_2_DCFDA) (LifeTechnologies, Gent, Belgium) and analyzed by flow cytometry (FACSCalibur). In the presence of ROS, the non fluorescent cell permeant DCFDA is converted in highly fluorescent 2',7'-dichlorofluorescein (DCF). 50 μM of H_2_O_2_ for 15 or 30 min were used as an inducer of ROS production (positive control). Relative intracellular ROS levels were depicted as mean fluorescence intensity (MFI).

### 3.8. Plumbagin Intracellular Uptake and Efflux

Exponentially growing Raji and U937 cells were exposed to 5 μM plumbagin. Plumbagin uptake was assessed by measuring plumbagin intracellular fluorescence from compound-loaded cells after different incubation times (15, 30, 45, 60, 90 and 120 min). At the end of these specific incubation times, cells were collected, centrifuged and re-suspended in fresh medium for further analysis. Efflux of plumbagin was evaluated in the following way, as previously described for doxorubicin [50]. After 30 min of incubation with plumbagin (5 μM), plumbagin-containing medium was removed and cells were re-suspended in fresh medium for recovery [50]. Fluorescence was evaluated immediately (T = 0 min) and after 15, 30, 60, and 90 min. Plumbagin fluorescence was evaluated at the indicated times by flow cytometry using a FACSCalibur, tuned at 488 nm, at standard pass filters; FL2 (FL2 = 585/42 nm). Data were recorded using the CellQuest software and further analyzed with FlowJo.

### 3.9. Analysis of GSH Content

Reduced (GSH) and oxidized (GSSG) glutathione measurements were performed using the GSH/GSSG-Glo™ Assay kit (Promega, Leiden, The Netherlands). Briefly, after treatment with plumbagin, 5 × 10^5^ cells are collected, centrifuged and resuspended in 1 mL of pre-warmed Hank’s Buffered Salt Solution (HBSS). Cells treated with BSO served as a positive control of depletion of GSH content [60]. A volume of 25 μL of the cell suspension is transferred into wells of a 96-well plate. An equivalent volume of appropriate lysis buffer is then added. GSH/GSSG-Glo assay is then performed following manufacturer’s instructions. The analysis of cellular GSH content was carried out by staining of the cells with o-phtalaldehyde (OPA), a permanent fluorescent probe. OPA is a direct tool that can interact with small thiol groups (e.g., GSH) in order to form adducts with them. Briefly, Raji and U937 cells were treated with 1 or 5 μM of plumbagin for 1 h. At the end of the incubation time, cells were washed with PBS and incubated with 50 μM OPA for 20 min. OPA fluorescence was evaluated by spectrofluorimetry (SpectraMax Gemini EM, Molecular Devices, Sunnyvale, CA, USA).

### 3.10. Statistical Analysis

Results from at least three independent experiments were analyzed for statistical significant differences using the Student’s *t*-test. They are expressed as the mean ± SD. *p*-values <0.05 (*), <0.01 (**) and <0.001 (***) were considered as statistically significant.

## 4. Conclusions

Plumbagin is a natural compound that exerts differential cytotoxicity towards leukemia cancer cells resulting from its modulatory activities on the cellular redox state, however the actual cellular targets of plumbagin in the redox control remain still under debate. ROS increase is commonly detected upon plumbagin treatment. Recently, several studies have pointed at the specific ability of plumbagin to modulate the intracellular thiols. These findings would imply the modulation of the intracellular thiolstat as relevant to trigger the anti-cancer effects of plumbagin, rather then the generation of ROS, which therefore would appear as a merely additional side effect, in the fact not essential for its anticancer activity. Our data seem to support this latter model (Table 3), as apoptosis induced by plumbagin in our sensitive (U937) and less sensitive (Raji) hematopoietic cancer cell models can be prevented only by antioxidants containing thiol species.

**Table 3:** Summary of the results. A horizontal arrow (➙) indicates that the antioxidant has no effect on the parameter observed compared to the control. A downward arrow (➘) indicates a decrease of the parameter observed compared to the control.

Cell Line	U937				Raji			
**Plumbagin model**	More sensitive (IC_50_ 24 h = 1 μM)				Less sensitive (IC_50_ 24 h = 5 μM)			
**Cell death**	Apoptosis							
**Plumbagin uptake**	Similar incorporation (up to 120 min)							
**Plumbagin efflux**	No efflux, fluorescence remains constant							
**ROS production**	Elevated				Moderate			
**GSH modulation**	GSH modulation depending on their respective IC_50_							
**Antioxidant classification**	Thiol group		Non-thiol group		Thiol group		Non-thiol group	
**Antioxidant**	DTT	NAC	Tiron	Trolox	DTT	NAC	Tiron	Trolox
**ROS production**	➘	➘➘	➙	➘	➙	➙	➙	➙
**Apoptosis**	➘	➘	➙	➙	➘	➘	➙	➙

There is evidence that plumbagin may directly interact with GSH, by likely a nucleophilic addition, which in turn may contribute to GSH depletion [22,45]. The small intracellular thiol GSH is paradigmatic for a huge group of additional and more complex intracellular thiols potentially targetable by plumbagin, which also includes many structural proteins and enzymes. Tubulin is among the proteins/enzymes known to be bound by plumbagin [61]. Remarkably, thiol modulation is particularly relevant also for the multi-step activation of the pro-apoptotic Bcl-2 family members [62,63,64]. Strong evidence suggests the modulation of specific Bax (Bcl-2-associated X protein) cysteine residues is critical for the acquisition of its suitable conformation, oligomerization and translocation/insertion into the mitochondrial membrane [65]. It would be relevant in the future to investigate any potential ability of plumbagin to directly interact and thereby modulate *i.e.*, Bax activation. Such interactions can further contribute to protein derivatization [45]. Glutathione-*S*-transferases, which control detoxification through the consumption of glutathione (GSH), may also be inactivated by plumbagin. This modulation is paralleled by ROS formation [66]. A previous analysis of GSTP1 level expression, in our lab revealed that the less plumbagin-sensitive Raji cells do not expressed GSTP1 proteins in contrast to the most sensitive U937 cell model here investigated [67,68]. These differential alterations may potentially provide additional hints to may identify the reason of such differences in ROS generation. Taken all together, our and other findings indicate the ability of plumbagin to may eventually lead to the modulation of important intracellular functions dominated by thiol modulation.
